# Scaling up of continuous-flow, microwave-assisted, organic reactions by varying the size of Pd-functionalized catalytic monoliths

**DOI:** 10.3762/bjoc.7.133

**Published:** 2011-08-23

**Authors:** Ping He, Stephen J Haswell, Paul D I Fletcher, Stephen M Kelly, Andrew Mansfield

**Affiliations:** 1 Department of Chemistry, University of Hull, Hull HU6 7RX, UK; 2 Pfizer Global Research & Development, Sandwich, Kent CT13 9NJ, UK

**Keywords:** continuous flow, microwave heating, monolith, scaling-up, Suzuki–Miyaura reaction

## Abstract

A product-scalable, catalytically mediated flow system has been developed to perform Suzuki–Miyaura reactions under a microwave heating regime, in which the volumetric throughput of a Pd-supported silica monolith can be used to increase the quantity of the product without changing the optimal operating conditions. Two silica monoliths (both 3 cm long), with comparable pore diameters and surface areas, were fabricated with diameters of 3.2 and 6.4 mm to give volumetric capacities of 0.205 and 0.790 mL, respectively. The two monoliths were functionalized with a loading of 4.5 wt % Pd and then sealed in heat-shrinkable Teflon^®^ tubing to form a monolithic flow reactor. The Pd-supported silica monolith flow reactor was then placed into the microwave cavity and connected to an HPLC pump and a backpressure regulator to minimize the formation of gas bubbles. The flow rate and microwave power were varied to optimize the reactant contact time and temperature, respectively. Under optimal reaction conditions the quantity of product could be increased from 31 mg per hour to 340 mg per hour simply by changing the volumetric capacity of the monolith.

## Introduction

Interest in flow based reaction chemistry has grown over recent years with the realization that such systems can offer greater control over reaction conditions, such as catalyst and heating contact time, which in turn lead to improved product selectivity and yield when compared to batch based methods [1–7]. Much of this work has focused on continuous-flow microreactor methodology for laboratory based organic synthesis, and has featured the development of inorganic and organic polymer based functionalized monolithic reactors that can operate at elevated temperatures and under high pressure [8–9]. Recently, application of magnetic nanoparticles as media that can be heated in an electromagnetic field, was reported to be ideal for use inside microfluidic fixed-bed reactors for chemical synthesis [10]. A new concept to build the catalytic membrane inside a microchannel reactor was demonstrated by Uozumi et al. [11], where carbon–carbon bond forming reactions of aryl halides and arylboronic acids under microflow conditions can be achieved quantitatively within 4 s residence time. However, the stability of the catalytic membrane was not discussed. Monolith based devices have shown good flow characteristics when coupled with the highly controlled surface properties associated with the formation of nano-, micro- and mesoporous structures, and they therefore represent ideal supports for reagents and catalysts where contact time and temperature can be spatially and temporally mediated [12–13]. To this end, the use of microwave heating in conjunction with microporous monolithic reactors has attracted some interest for small-scale synthesis under continuous-flow conditions [14–16]. One obvious problem, however, when using microwaves to heat solvents/reagents and surface-functionalized monoliths in a flow microreactor, is the achievement of an efficient coupling of the microwave energy, which will be a function of both the absorbing species present and of the penetration depth of microwave irradiation into the reaction zone [17]. This is especially important in flow systems where the reactants are present in the irradiation chamber for a short period of time [18–19]. Therefore, the application of microwave chemistry to scalable, continuous-flow processes, with commercially available microwave equipment and suitable flow instrumentation, is becoming increasingly important [7,20]. Finally, the high surface-to-volume ratio and spatial and temporal control over the reactants and products, without the need for additional optimization, is of considerable interest [7,21–23] as these factors promise to increase the quantity of product to desirable levels whilst maintaining the intrinsic benefits of the reaction geometry offered when using microreactor methodology.

In this work we report a simple and effective approach for achieving volumetric scalability in a flow reaction system through the use of Pd-supported silica-based monolithic reactors coupled with microwave heating. The practicality of this approach will be demonstrated using Suzuki–Miyaura reactions in which the Pd-supported silica-monolith catalysts exhibit excellent activities and the doubling of the monolith diameter, thus operating at four times the volumetric flow rate, increases product output without any observable change in the reaction conversion.

## Results and Discussion

### Synthesis of silica monolith and Pd-supported silica monolith catalyst

The reaction parameters, such as polymer concentration, acid strength, water content, amount of silicon alkoxide, reaction temperature and reaction time, all have an important impact on the physical properties of the silica monoliths prepared. Silica monoliths used as catalyst supports require not only a high surface area to maximize their catalytic activity, but also a high permeability to achieve good flow characteristics and enable fast mass transfer from the flowing reaction solutions to the catalytic surface. In addition, they must be mechanically strong enough to withstand the pressures required to drive fluid through the monolithic structure at the required flow rate. Silica monoliths were synthesized from PEO, tetraethoxysilane and nitric acid as supports for the Pd catalyst, as based on the results of previous studies [24]. Silica monoliths, i.e., monolith-3.2 and monolith-6.4 (diameters of 3.2 mm and 6.4 mm respectively), with two different diameters were synthesized using the same procedure, leading to structures with comparable surface characteristics but with different volumes for the solution-accessible connected pores. Characterization of these monoliths indicated that a 2-fold increase in the monolith diameter had little influence on the physical characteristics of the monoliths (see Table 1, entries 1 and 3, 2 and 4), except the total volume that was increased by a factor of almost four, as expected. In addition, the loading of metal particles within monoliths had no effect on either the nm-scale or µm-scale pore structures (see Table 1, entries 1 and 2, 3 and 4, also see Supporting Information File 1, Figure S1).

**Table 1:** The main characteristics of monoliths characterized by N_2_ adsorption at 77 K.^a^

Entry	Monolith	*D*_N2_	*S*_BET_	*V*_N2_	*V*_water_	φ_t_
		(nm)	(m^2^ g^−1^)	(cm^3^ g^−1^)	(mL)	

1	monolith-3.2	16.0	164	0.70	0.205	0.85
2	Pd-monolith-3.2	15.9	169	0.67	0.202	0.84
3	monolith-6.4	16.1	161	0.73	0.791	0.82
4	Pd-monolith-6.4	16.0	166	0.67	0.790	0.82

^a^*D*_N2_, *S*_BET_ and *V*_N2_ are the pore diameter, specific surface area and pore volume, respectively, as determined by N_2_ adsorption at 77 K. *V*_water_ is the total volume of the monoliths as measured by the adsorption of water at room temperature. φ_t_ is the total porosity as determined by equation (*W*_M_ − *W*_T_)/*dlr*^2^π, here *W*_T_ and *W*_M_ are the weights of the dry and water filled monolith respectively, *d* is the density of water and *l* and *r* are the overall length and radius of the cylindrical monoliths. The palladium loading for entries 2 and 4 was ca. 4.5 wt %.

**Figure 1:**
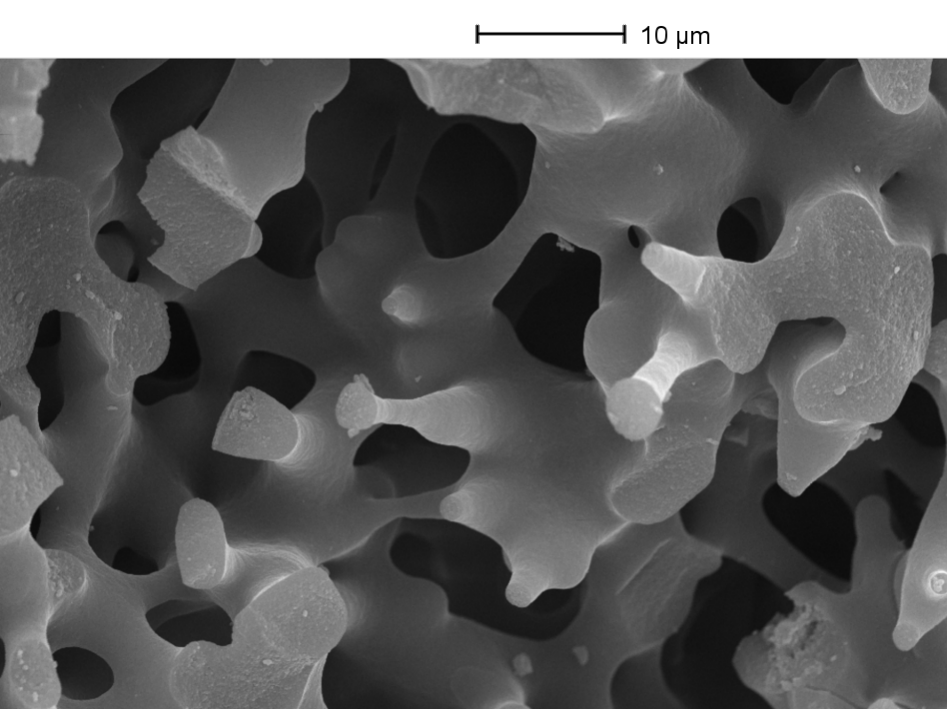
SEM image of silica monolith.

According to IUPAC [24] the measurements obtained from N_2_ adsorption and desorption isotherms indicate a type H2 hysteresis, which is consistent with the disordered mesoporous structure seen in the micrograph shown in Figure 1 (also see Supporting Information File 1, Figure S2)

### Effect of Pd precursor on the activity of the Pd-monolith catalyst

**Scheme 1:**
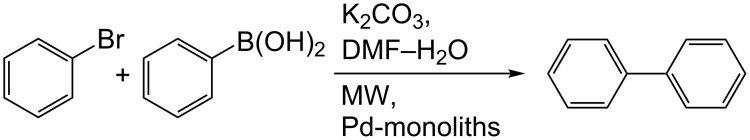
Suzuki–Miyaura reaction of bromobenzene with phenylboronic acid.

The Suzuki–Miyaura reaction is a widely used method in organic synthesis for the selective formation of aryl–aryl carbon–carbon bonds in the synthesis of high-value fine chemicals and intermediates in the pharmaceutical industry. This reaction requires a metal catalyst, such as palladium, in both homogeneous and heterogeneous reactions. In this study, the Suzuki–Miyaura reaction of bromobenzene with phenylboronic acid (Scheme 1) was initially used as a model heterogeneously catalyzed reaction for the evaluation of Pd-monolith activity under continuous flow conditions with microwave heating.

The silica monoliths were impregnated with a range of Pd precursors, namely Na_2_PdCl_4_, Pd(OAc)_2_, Pd(dba)_2_ and Pd(NO_3_)_2_, by a standard method described previously for the preparation of Pd-monoliths. The Pd-monolith-3.2 catalysts were evaluated using the previously optimized solvent and basic reaction conditions [3–4]. The results of this study (Table 2, entries 1–4) indicate that, whilst all the Pd-monolith catalysts contain the same amount of palladium (around 4.5 wt %), their catalytic activity differs significantly even for similar reaction temperatures and contact times, showing a significant effect of the palladium precursor on the catalyst activity. The Pd-monolith catalyst that was synthesized from a Na_2_PdCl_4_ precursor showed the best activity and was therefore used as the Pd precursor for the preparation of a Pd-monolith catalyst to be employed in further investigations. As expected, reducing the reaction temperature and decreasing the catalyst contact time (see Table 2, entries 4–7) resulted in a corresponding reduction of the product yield.

**Table 2:** Reactivity of Pd-monolith-3.2 synthesized using different Pd precursors in the Suzuki–Miyaura reaction between bromobenzene and phenylboronic acid under continuous flow conditions.^a^

Entry	Pd precursor	MW power	Temperature	Flow rate	Contact time	Conversion
		(W)	(°C)	(μL min^−1^)	(min)	(%)

1	Pd(OAc)_2_	8	123	20	10	72
2	Pd(dba)_2_	8	123	20	10	55
3	Pd(NO_3_)_2_	15	123	20	10	28
4	Na_2_PdCl_4_	5	123	20	10	97
5	Na_2_PdCl_4_	3	99	20	10	70
6	Na_2_PdCl_4_	10	116	40	5	66
7	Na_2_PdCl_4_	5	109	40	5	45

^a^All Pd-monolith catalysts have a Pd-loading of ca. 4.5 wt %. Conversions were determined using GC–MS versus internal standard. The main byproducts (1–3%) were formed by the debromination of halide reactants.

### Comparison of activity between Pd-monolith-3.2 and Pd-monolith-6.4

**Figure 2:**
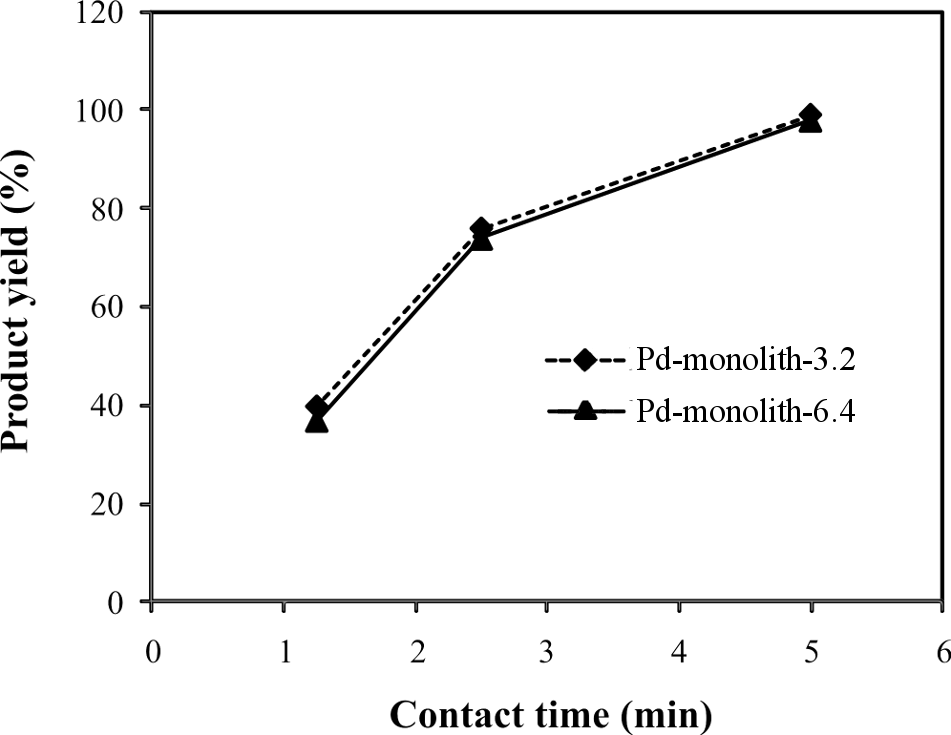
Reactivity of the Pd-monolith-3.2 and Pd-monolith-6.4 for the Suzuki–Miyaura reaction between bromobenzene (0.1 M) and phenylboronic acid (0.12 M): The relationship of product yield with contact time.

The main aim of this work is to develop a methodology to scale up the rate of product formation without a loss in the intrinsic reaction performance, by using a continuous-flow, microwave-assisted, Pd-supported silica-monolith reactor. The Pd-monolith-3.2 and Pd-monolith-6.4 (both with the same length of 3 cm) were used to perform the model reaction (1) to demonstrate this methodology. The total pore volume accessible to the solution, determined by adsorption of water, was 0.20 mL for Pd-monolith-3.2 and 0.79 mL for Pd-monolith-6.4, which represents an almost 4-fold volume increase for the larger Pd-monolith-6.4. The activities of both monoliths for the Suzuki–Miyaura reaction (Scheme 1) are shown in Figure 2. It can be seen that Pd-monolith-6.4 produces a very similar percentage yield of product to that obtained using the Pd-monolith-3.2 under four times the flow rate to keep the same catalyst contact time. This observation is suggestive of virtually identical intrinsic properties for both monoliths in terms of flow rate and reaction conversion.

**Figure 3:**
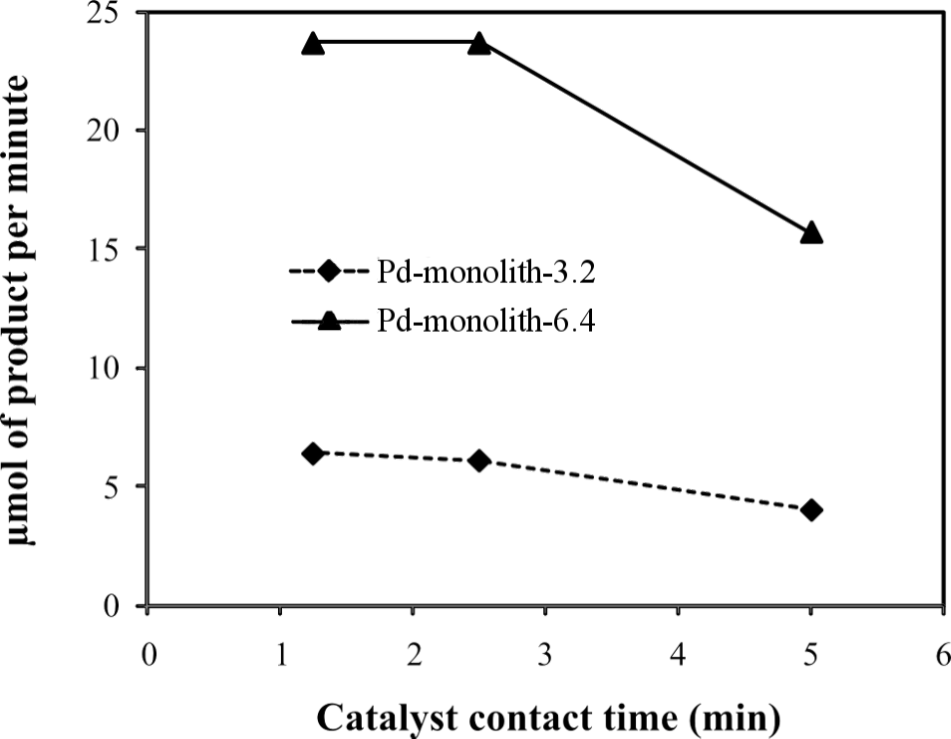
Reactivity of the Pd-monolith-3.2 and Pd-monolith-6.4 for the Suzuki–Miyaura reaction between bromobenzene (0.1 M) and phenylboronic acid (0.12 M): The dependence of micromoles of product obtained on contact time.

It can also be seen (Figure 3) that the rate of product formation scales up as expected, i.e., the larger catalyst monolith produces four times as much product compared to the smaller catalyst monolith under equivalent reaction conditions. However, it is also evident from the data that whilst shorter catalyst contact times (corresponding to higher solution flow rates) produce an increase in the rate of product formation, this increased rate of product formation is at the expense of reduced reagent conversion.

This methodology was also used to test Suzuki–Miyaura reactions with a variety of substrates, as shown in Table 3. It can be seen that this scale-up strategy also works very well, with the amount of product obtained with the Pd-monolith-6.4 being four times greater than that obtained with the Pd-monolith-3.2, under these conditions. Most reagents generated an excellent reaction conversion of the desired coupling product (see Supporting Information File 1, Figures S3, S4 and S5), even in the case of chlorobenzene, which is a notably poor substrate for the Suzuki–Miyaura reaction (entries 6 and 12). The Suzuki–Miyaura reaction between bromobenzene and 4-bromobenzaldehyde with a higher concentration of reactants, i.e., 0.3 M, was also performed with the Pd-monolith-6.4 catalyst to evaluate the conversion ability. The reaction conversion was found to be high, i.e., 87–89 %, under these modified conditions.

**Table 3:** Reactivity of Pd-monoliths with different diameters, in the Suzuki–Miyaura reaction between various reactants under continuous-flow conditions.^a^

Entry	Catalyst	Flow rate	Halide	Boronic acid	Product	Conversion
		(μL min^−1^)				(%)

1	Pd-monolith-3.2	40	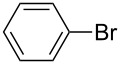	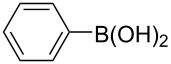	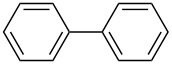	99
2	Pd-monolith-3.2	20	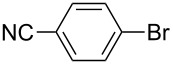	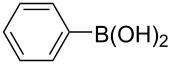	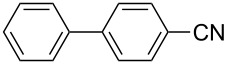	100
3	Pd-monolith-3.2	30	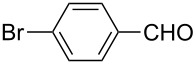	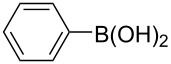	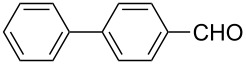	95
4	Pd-monolith-3.2	20	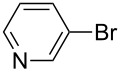	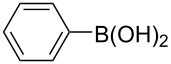	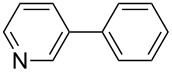	65
5	Pd-monolith-3.2	20	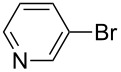	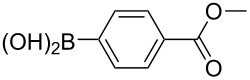	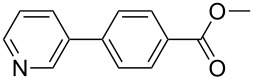	60
6	Pd-monolith-3.2	20	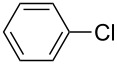	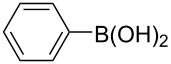	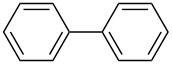	99

7	Pd-monolith-6.4	160	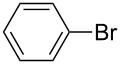	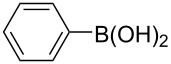	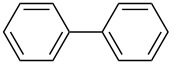	99
8	Pd-monolith-6.4	80	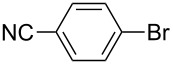	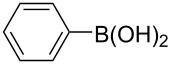	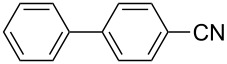	99
9	Pd-monolith-6.4	120	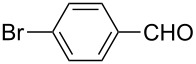	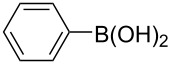	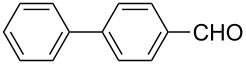	95
10	Pd-monolith-6.4	80	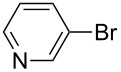	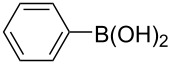	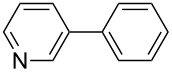	65
11	Pd-monolith-6.4	80	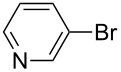	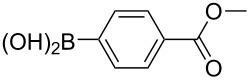	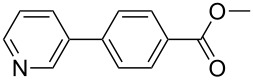	59
12	Pd-monolith-6.4	80	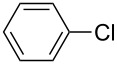	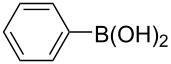	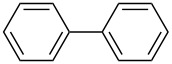	98

^a^MW power used was 5–10 W for Pd-monolith-3.2 and 1–2 W for Pd-monolith-6.4 to a maintain reaction temperature of 125–130 °C. The backpressure valve was set up 75 psi for Pd-monolith-3.2 and 45 psi for Pd-monolith-6.4, respectively. The reaction conversion was determined by GC–MS with an internal standard and the main byproduct (1–3%) was formed by debromination of halide reactants.

**Figure 4:**
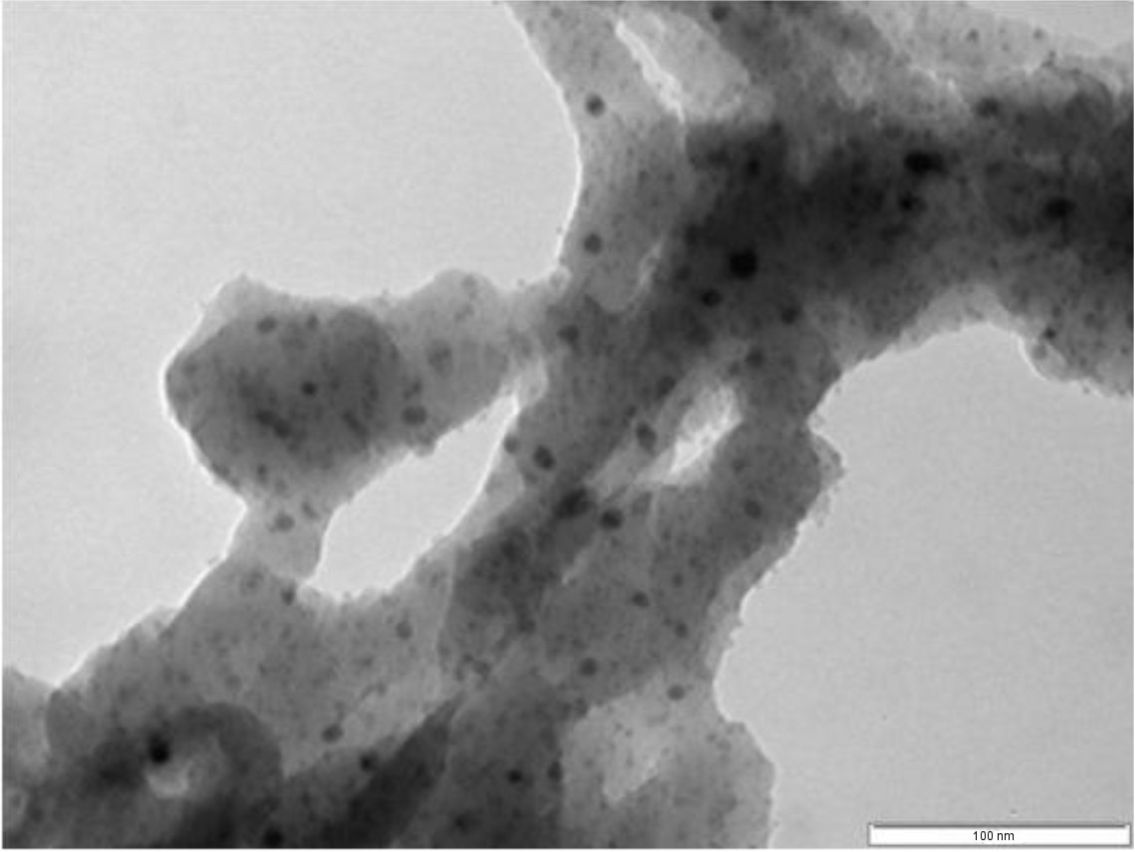
TEM image of Pd-monolith catalyst (scale bar: 100 nm).

The high catalytic activity of the Pd-monolith catalysts in Suzuki–Miyaura reactions can be attributed to the following three factors. First is the high dispersion of small Pd particles over the substrate surface within the monolith mesopores. The TEM image (Figure 4) shows that the catalyst sample incorporates metal particles, distributed over the substrate surface, with two different sizes: Small crystallites with dimensions of less than 2 nm (majority), and large crystallites with diameters of around 10 nm. Second is the large surface-to-volume ratio of the monoliths. The values of the surface-area-to-volume ratio for the microchannels typically range from 10,000 to 50,000 m^2^/m^3^, as a consequence of their decreased size. Based on BET characterization, the surface-area-to-volume ratio generated within the Pd-monolith-3.2 reactor was estimated to be 2.5 × 10^8^ m^2^/m^3^, which contributed greatly to the promotion of the reaction. The final factor relates to the combination of microwave heating and palladium nanoparticles. Kappe et al. found that smaller particles are more active in traditional heating whereas bigger particles perform better in microwave heating [25]. The monolithic structure used in this work takes advantage of both these characteristics by having more reactive, nano-sized, Pd particles located within a strongly microwave-absorbing, meso-size, silica structure. Because it was difficult to measure the temperature inside the monolith, the outlet temperature measured by fiber probe was used, which gave a difference of at least 20 °C between the outlet temperature and the temperature of the outer surface of the monolith, as measured using the installed IR sensor. It was found that reaction conversion was only 40–50% with oil bath heating.

With supported Pd catalysts, leaching of palladium is always an issue of concern in terms of catalyst performance, cost and recovery. Recent papers have shown that leaching from palladium catalysts is in the order of 1 to several tens of ppm [6,10,19,25] and that recovery of this palladium could be achieved with a scavenger column [6]. The tendency for leaching of palladium metal from the Pd-monoliths was measured through an ICP–OES analysis to determine the Pd concentration in the washing liquid, which immediately followed the first flow reaction experiment. This was achieved by pumping DMF/H_2_O (3:1) solvent through the Pd-monolith at 0.1 mL/min for 20 min. For the Na_2_PdCl_4_ based monolithic catalyst, the amount of palladium present in the washing liquid was found to be as little as 74 ppb (Pd-monolith-3.2), corresponding to a loss of only 0.000011% of the initial amount of palladium added to the monoliths. The amount of palladium present in the final reaction sample was found still to be less than 100 ppb. This finding suggests that there is a highly specific and strong interaction between the impregnated metal nanoparticles and the monolith support surface, possibly through a combination of hydrogen bonding, ionic interactions and substitution of Cl by silanol groups present on the monolith surface, resulting in highly stable nanoparticle fixation [26–27]. The presence of a strong specific metal/support interaction is also supported by observations made during the impregnation process, where PdCl_4_^2−^ uptake in the silica monolith body was seen to be fast, with the monolith turning a stable dark brown color after several hours. In contrast, the Pd(NO_3_)_2_ salt gave the monolith a lighter coloration, much more slowly, and was easily washed away. The PdCl_4_^2−^ based Pd-monolith catalyst was in fact used for several runs (i.e., 6 runs representing 15–20 hours) with no deactivation being observed when the catalyst was washed with DMF, water or DMF/H_2_O (3:1) after completion of each run.

## Conclusion

It has been demonstrated that the combination of Pd-functionalized silica monolithic reactors with microwave heating results in a high percentage yield of the desired reaction products for Suzuki–Miyaura reactions under flow conditions. Yields can be scaled-up by increasing the diameter of the catalytic monolith used. The cylindrical catalyst monoliths were of a constant length, but of variable diameter and were produced to give the same intrinsic monolith activity and permeability properties, when operating under the same conditions of temperature and catalyst contact time. In this way the product formation rate scales quantitatively with the square of the catalyst monolith diameter. However, at least one alternative approach can be envisaged, i.e., changing the monolith length for scaling up whilst maintaining the required intrinsic properties. It is worth considering the relative advantages and disadvantages of these two possible approaches in light of the work presented. Increasing the diameter of a fixed length monolith, the scale-up method as used here, offers the advantage that the pressure drop required to produce a certain flow rate decreases with increasing diameter. However, as microwave penetration is necessary to obtain reliable heating characteristics, there will come a point at which the monolith diameter will become larger than the penetration depth of the microwaves (estimated to be 4 cm), which will lead to an unheated, cold “core”. In addition for disc-shaped monoliths, where diameters are larger than the length, there will also come a point where the mechanical strength of the monolith will be a limitation with respect to the pressure drop required for flow. On the other hand, increasing the length of a fixed diameter monolith in order to achieve this scale-up offers the advantage that uniform microwave penetration/heating can be maintained. In addition, the catalyst contact time could be extended by increasing the length. The disadvantage, however, to this approach is related to the pressure drop required to produce the required flow rate, which will increase proportionally with the length. Hence, the mechanical strength of the monolith structure, i.e., the strength to resist collapse of the pores and/or the monolith casing material, ultimately limits the maximum length achievable.

## Experimental

### Materials

The reagents and solvents bromobenzene (99%), 4-bromobenzonitrile (99%), 4-bromobenzaldehyde (99%), 3-bromopyridine (99%), chlorobenzene (99%), phenylboronic acid (97%), 4-carboxyphenylboronic acid (97%), poly(ethylene oxide) (PEO) with average relative molar mass of 100 kDa, tetraethoxysilane (TEOS), *N,N*-dimethylformamide (99%, DMF), dichloromethane (99%, DCM), ammonium hydroxide (5 N) and nitric acid aqueous solutions (1 N) were purchased from Aldrich. All reagents were used as obtained, without further purification. Heat shrinkable Teflon^®^ tubes (wall thickness 0.1 and 0.3 mm before and after shrinkage) with a shrinkage ratio of 2:1 were purchased from Adtech Polymer Engineering Ltd. (UK).

### Synthesis of silica monolith supports

Silica based monoliths were prepared using a sol–gel process described in the literature [24]. The desired amount of PEO was added to an aqueous solution of nitric acid and the resultant mixture was cooled in an ice bath and stirred until a homogeneous solution formed. TEOS was then added to the reaction mixture, which was stirred vigorously in the ice bath for 30 min to form a transparent solution. Subsequently, the solution was poured into a plastic mould (diameter 4.8 mm and length 6 cm for monolith-3.2; and diameter 8.2 mm and length 5 cm for monolith-6.4). Both ends of the plastic mould were then closed and the sealed tube was incubated in an oven at 40 °C for 3 days, during which time a wet, semi-solid, gel monolith was formed. Approximately 20% shrinkage occurred during this gel formation, which allowed easy removal of the wet gel monoliths from the plastic tube moulds. The wet gel monoliths were washed with copious amounts of water to remove any residues and then transferred to a 10 times larger volume of 0.5 M NH_4_OH aqueous solution in an autoclave, where it was incubated at 80 °C for 24 h. The monoliths were again washed with copious amounts of water before drying in an oven at 90 °C for 24 h. Finally, the monoliths were calcined at 550 °C for 3 h (heating rate: 2 °C/min) in an air flow to remove the remaining PEO and form white silica-monolith rods (diameters 3.2 and 6.4 mm respectively) that were then cut to 3 cm long monoliths.

### Preparation of Pd-supported silica-monolith catalyst (Pd-monolith)

An aqueous solution of 200 μL containing 0.017 g Na_2_PdCl_4_ (theoretical Pd loading 5.0 wt %) was adsorbed onto the monoliths, dried at 90 °C and calcined at 550 °C for 3 h (temperature ramp: 2 °C min^−1^) under a flow of air, followed by reduction in a H_2_ (10%)/N_2_ stream at 340 °C for 3 h (heating rate: 2 °C) to produce a black Pd-monolith rod with Pd loading of approximately 4.5 wt % as determined by ICP–OES (Perkin Elmer Optima 5300DV). The Pd-monolith rod obtained was then clad in a heat-shrinkable Teflon^®^ tube with a glass connector at each end. The assembly was heated in a furnace up to 330 °C until the monolith was sealed within the Teflon^®^ tube to form a flow Pd-monolith reactor system.

### Sample characterization

Scanning electron microscopy (SEM) images were obtained by means of a Cambridge S360 scanning electron microscope operated at 20 kV. Each sample was sputter coated with a thin layer of gold–platinum (thickness approximately 2 nm) by a SEMPREP 2 Sputter Coater (Nanotech Ltd.). Transmission electron microscopy (TEM) was carried out on a JEOL-2010 operating at 200 kV. The BET surface area and nm-scale pore-size distribution were obtained by measuring N_2_ adsorption and desorption isotherms at 77 K by means of a Micromeritics Surface Area and Porosity Analyzer. The pore volume and pore size distributions of the nm-scale pores within the monoliths were evaluated from the isotherms within the BJH (Barrett-Joyner-Halenda) model. The palladium content in the monoliths and washing liquid was determined by ICP–OES. Determination of the µm-scale porosity φ_t_ (which determines the monolith permeability) was determined from the equation (*W*_M_ − *W*_T_)/*dlr*^2^π, where *W*_T_ and *W*_M_ were the weights of the dry and water filled monolith respectively, *d* was the density of water, *l* and *r* were the overall length and radius of the cylindrical monolith. The µm-scale pore size was determined from SEM measurements.

### Activity measurements

The experimental setup is shown schematically in Supporting Information File 1, Figure S6. The 30 mm long Pd-monolith reactor with a diameter of either 3.2 mm (Pd-monolith-3.2) or 6.4 mm (Pd-monolith-6.4) was positioned in the cavity of a Discover microwave system (CEM Ltd.) with the capability of delivering 0–300 W of microwave power at 2.45 GHz with mono-mode operation. The microwave cavity was fitted with an infrared sensor to monitor the temperature of the external surface of the monolith catalyst. A reactant solution containing an aryl halide (0.1 M), arylboronic acid (0.12 M), K_2_CO_3_ (0.3 M) in DMF/H_2_O (3:1) solvent was pumped through the reactor with an HPLC pump, and a backpressure valve (45–75 psi) was used to minimize the formation of gas bubbles (see Supporting Information File 1, Figure S6). The residence times of the reactants within the catalytic monoliths were determined based on the known monolith and pore volume and from the different flow rates. Product samples were collected at defined flow periods during a reaction run, weighed and a known amount of dodecane was added to the individual samples as an internal standard. Samples were treated with 1 M aqueous NaOH to remove unreacted arylboronic acid and extracted with DCM. The remaining organic material was then washed three times with distilled water, collected and dried over MgSO_4_. Individual samples were analyzed using GC–MS (Varian 2000) as described in literature [3–4].

## Supporting Information

The Supporting Information File contains six parts, Figure S1: SEM image of Pd-monolith; Figure S2: BET characterization; Figure S3: GC–MS chromatogram for Suzuki–Miyaura reaction of bromobenzene and phenylboronic acid; Figure S4: GC–MS chromatogram for Suzuki–Miyaura reaction of 4-bromobenzaldehyde and phenylboronic acid; Figure S5: GC–MS chromatogram for Suzuki–Miyaura reaction of 4-bromobenzonitrile and phenylboronic acid; Figure S6: Schematic diagram of the setup for continuous-flow, microwave-assisted Suzuki–Miyaura reactions.

File 1 Additional material.

## References

[1] Vankayala B K, Löb P, Hessel V, Menges G, Hoffmann C, Metzke D, Krtschil U, Kost H-J (2007). Int J Chem React Eng.

[2] de Mas N, Günther A, Kraus T, Schmidt M A, Jensen K F (2005). Ind Eng Chem Res.

[3] He P, Haswell S J, Fletcher P D I (2004). Lab Chip.

[4] He P, Haswell S J, Fletcher P D I (2004). Appl Catal, A.

[5] Glasnov T N, Kappe C O (2010). Adv Synth Catal.

[6] Mennecke K, Kirschning A (2009). Beilstein J Org Chem.

[7] Razzaq T, Kappe C O (2010). Chem–Asian J.

[8] Comer E, Organ M G (2005). Chem–Eur J.

[9] Gömann A, Deverell J A, Munting K F, Jones R C, Rodemann T, Canty A J, Smith J A, Guijt R M (2009). Tetrahedron.

[10] Ceylan S, Friese C, Lammel C, Mazac K, Kirschning A (2008). Angew Chem, Int Ed.

[11] Uozumi Y, Yamada Y M A, Beppu T, Fukuyama N, Ueno M, Kitamori T (2006). J Am Chem Soc.

[12] Svec F, Huber C G (2006). Anal Chem.

[13] Preinerstorfer B, Bicker W, Lindner W, Lämmerhofer M (2004). J Chromatogr, A.

[14] Smith C J, Smith C D, Nikbin N, Ley S V, Baxendale I R (2011). Org Biomol Chem.

[15] Kunz U, Kirschning A, Wen H-L, Solodenko W, Cecilia R, Kappe C O, Turek T (2005). Catal Today.

[16] Nikbin N, Ladlow M, Ley S V (2007). Org Process Res Dev.

[17] Kappe C O, Dallinger D, Murphree S S (2009). Practical Microwave Synthesis for Organic Chemists – Strategies, Instruments, and Protocols.

[18] Comer E, Organ M G (2005). J Am Chem Soc.

[19] Shore G, Morin S, Organ M G (2006). Angew Chem, Int Ed.

[20] Singh B K, Kaval N, Tomar S, Van der Eycken E, Parmar V S (2008). Org Process Res Dev.

[21] Glasnov T N, Kappe C O (2007). Macromol Rapid Commun.

[22] Mason B P, Price K E, Steinbacher J L, Bogdan A R, McQuade D T (2007). Chem Rev.

[23] Pennemann H, Watts P, Haswell S J, Hessel V, Löwe H (2004). Org Process Res Dev.

[24] Fletcher P D I, Haswell S J, He P, Kelly S M, Mansfield A J (2010). Porous Mater.

[25] Mennecke K, Cecilia R, Glasnov T N, Gruhl S, Vogt C, Feldhoff A, Vargas M A L, Kappe C O, Kunz U, Kirschning A (2008). Adv Synth Catal.

[26] Bronstein L M, Polarz S, Smarsly B, Antonietti M (2001). Adv Mater.

[27] Kosslick H, Mönnich I, Paetzold E, Fuhrmann H, Fricke R, Müller D, Oehme G (2001). Micro Meso Mater.

