# Supplementary material for: Scaling up of continuous-flow, microwave-assisted, organic reactions by varying the size of Pd-functionalized catalytic monoliths
Source: Beilstein J Org Chem. 2011 Aug 23;7:1150–7. doi: 10.3762/bjoc.7.133 (PMC3170192; doi:10.3762/bjoc.7.133)
Supplement: File 1 — Additional material. [file Beilstein_J_Org_Chem-07-1150-s001.pdf]

Supporting information

for

Scaling up of continuous-flow, microwave-assisted, organic reactions by varying the size of Pd-functionalized catalytic monoliths

Ping He[1], Stephen J. Haswell[1,*], Paul D. I. Fletcher[1], Stephen M. Kelly[1] and Andrew Mansfield[2]

Address: [1]Department of Chemistry, University of Hull, Hull HU6 7RX, UK and [2]Pfizer Global Research & Development, Sandwich, Kent CT13 9NJ, UK

Email: Stephen J. Haswell - s.j.haswell@hull.ac.uk

* Corresponding author

Additional material

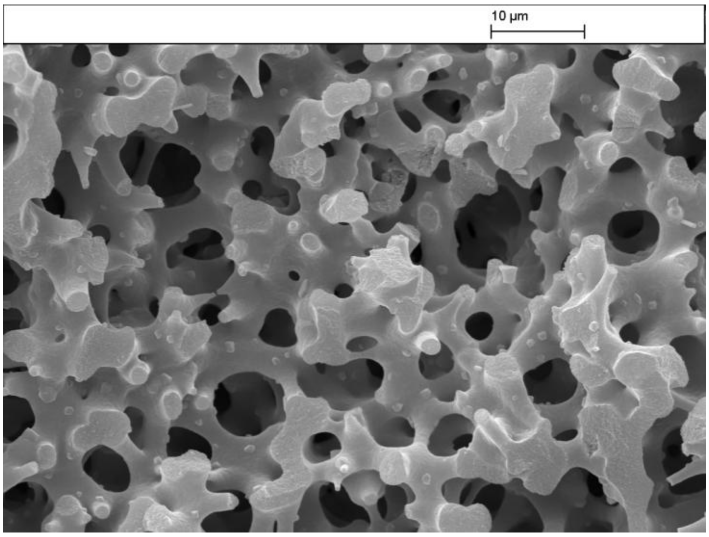

Figure S1: SEM image of Pd-Monolith

(a) (b)

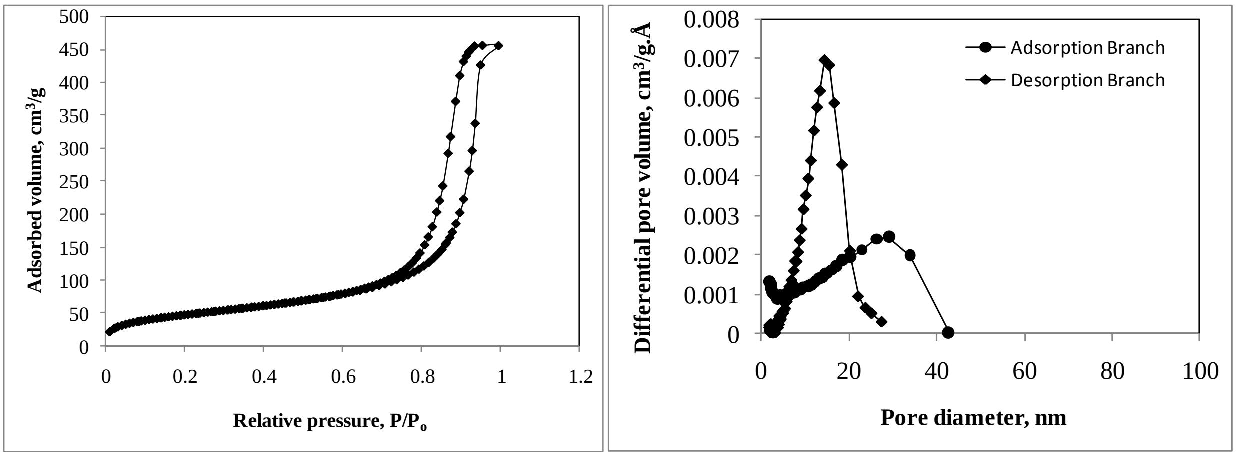

Figure S2: BET characterization

(a) N_2 adsorption/desorption at 77 K in monolith; (b) pore size distribution obtained from adsorption and desorption branch.

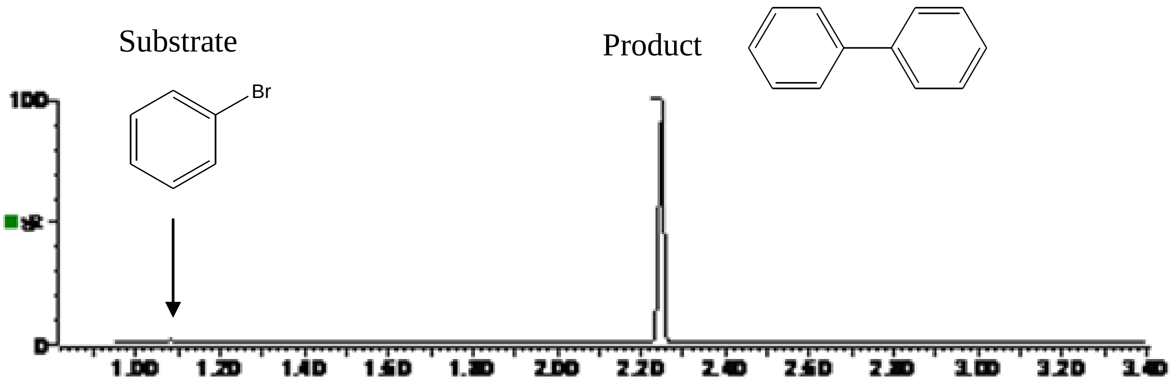

Figure S3: GC–MS chromatogram for Suzuki–Miyaura reaction of bromobenzene and phenylboronic acid

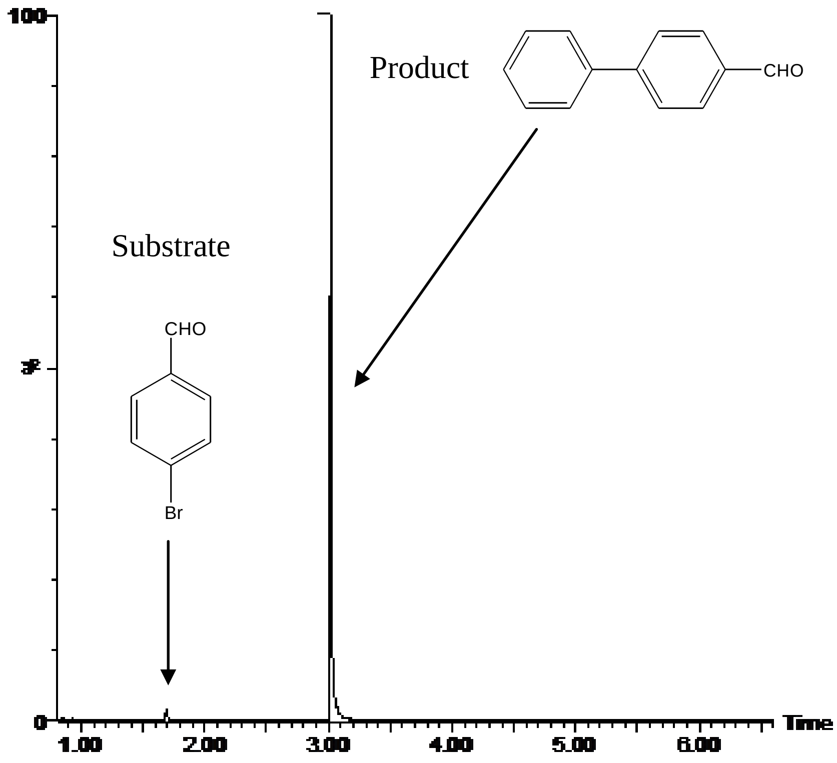

Figure S4: GC–MS chromatogram for Suzuki–Miyaura reaction of 4-bromobenzaldehyde and phenylboronic acid

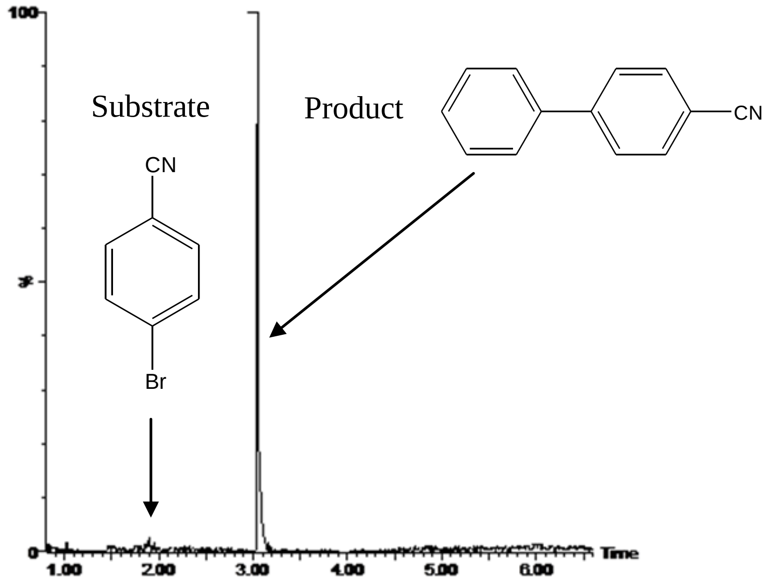

Figure S5: GC–MS chromatogram for Suzuki–Miyaura reaction of 4-bromobenzonitrile and phenylboronic acid

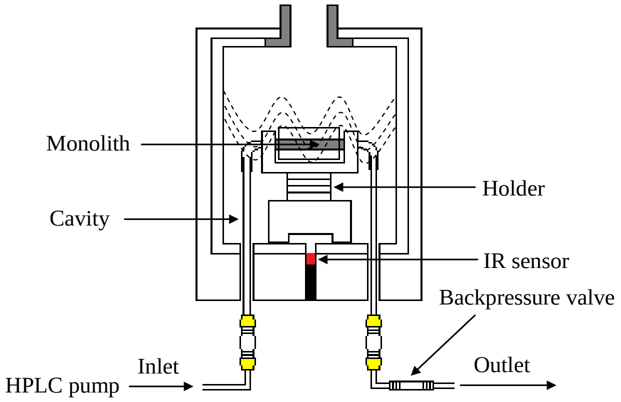

Figure S6: Schematic diagram of the setup for continuous-flow microwave-assisted Suzuki reactions

An HPLC pump was used for pumping the reaction mixture through the Pd-monolith reactor, which was well positioned in a home-made holder within the cavity of a Discovery microwave. An infrared sensor was fitted in the bottom of the cavity to monitor the temperature of the external surface of the monolith reactor.
